# A Rare Combination of Patellar Sleeve Fracture, Tendon Avulsion, and Retinacular Disruption in a Child: Diagnostic and Operative Insights

**DOI:** 10.7759/cureus.108404

**Published:** 2026-05-06

**Authors:** Ali Tariq, Muhammad Mubeen Bashir, Mahrukh Azhar, Eswara Murthy, William Gaine

**Affiliations:** 1 Orthopaedic Surgery, Mayo Hospital, Castlebar, IRL; 2 Orthopaedics and Trauma, Sligo University Hospital, Sligo, IRL; 3 Palliative Care, Mayo Hospice, Castlebar, IRL; 4 Orthopaedic Surgery, Sligo University Hospital, Sligo, IRL

**Keywords:** case report, extensor mechanism, patellar sleeve fracture, pediatric knee injury, retinacular disruption, tendon avulsion

## Abstract

A 13-year-old boy presented with acute right knee pain, significant effusion, and a complete loss of active extension following a football injury. Radiographs demonstrated patella alta and increased density within the infrapatellar fat pad. Intraoperative exploration revealed a rare "triple injury" triad: a displaced inferior pole patellar sleeve fracture, a distal patellar tendon avulsion from the tibial insertion, and near-complete medial and lateral retinacular disruption.

Surgical reconstruction was performed using suture-tape fixation with anchors for the patellar sleeve, tendon reinsertion utilizing Krakow sutures with screw augmentation, and primary retinacular repair. Following a structured postoperative rehabilitation program, the patient achieved an excellent functional outcome with restored full range of motion. This case highlights a complex multilevel extensor mechanism disruption in a skeletally immature patient and the surgical approach used for anatomical restoration.

## Introduction

Extensor mechanism injuries in children are rare, comprising less than 1% of pediatric fractures [1,2]. Most involve isolated elements such as patellar sleeve fractures [1,3] or tibial tubercle avulsions [4]. Combined bone and tendon disruptions are exceedingly rare [5,6], often resulting from high-energy eccentric loading of the quadriceps while the knee is flexed [7-9]. We present a unique case of inferior patellar sleeve fracture, distal tendon avulsion, and bilateral retinacular disruption, emphasizing diagnostic and operative considerations.

## Case presentation

A 13-year-old boy sustained a twisting football injury with immediate pain, swelling, and inability to extend the right knee. He experienced immediate pain, swelling, and an inability to perform active knee extension. Examination revealed knee joint effusion and loss of active extension.

**Figure 1:**
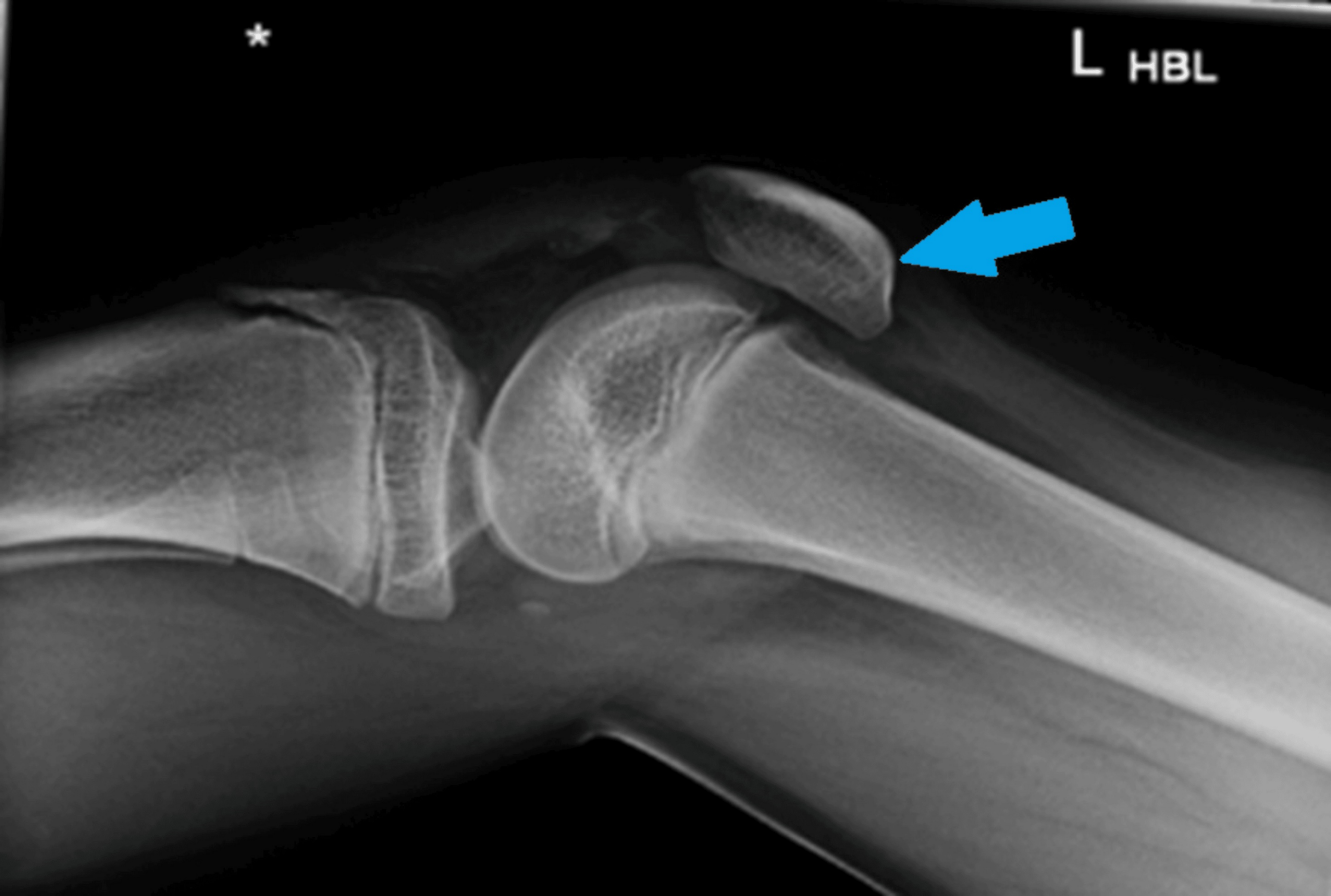
X-ray lateral view on initial presentation. The blue arrow shows patella alta and an abnormal Insall-Salvati ratio.

Similar presentations of distal patellar tendon avulsions often require a high index of suspicion when radiographs appear subtly altered [2,4]. Radiographs showed patella alta, infrapatellar soft-tissue density, and an Insall-Salvati ratio of more than 1.2 (normally between 0.8 and 1.2) (Figure 1) [1,2]. Urgent surgery was performed without a preoperative MRI due to clear clinical signs of extensor disruption [10].

**Figure 2:**
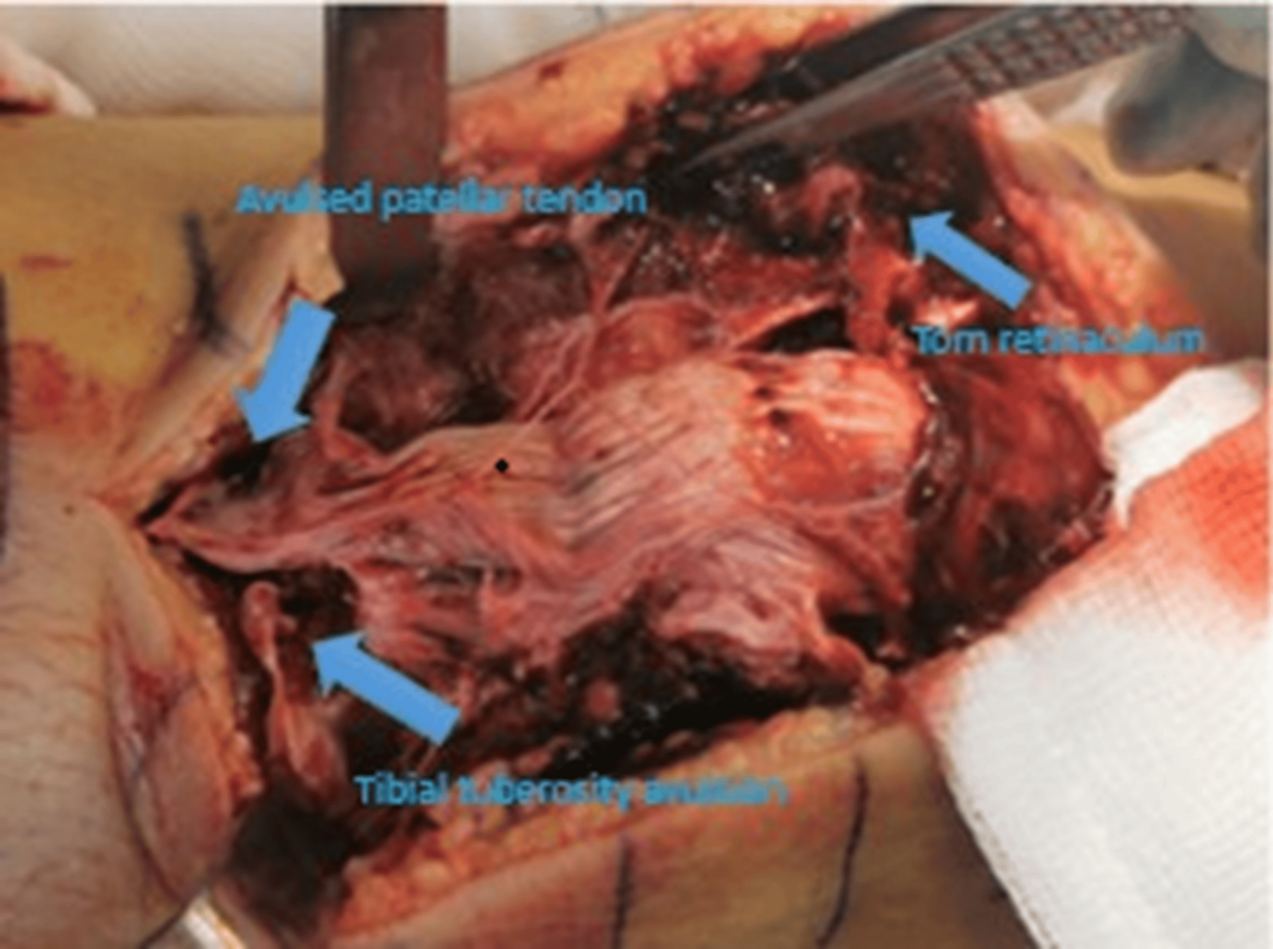
Intraoperative image showing extensive disruption of extensor mechanism.

**Figure 3:**
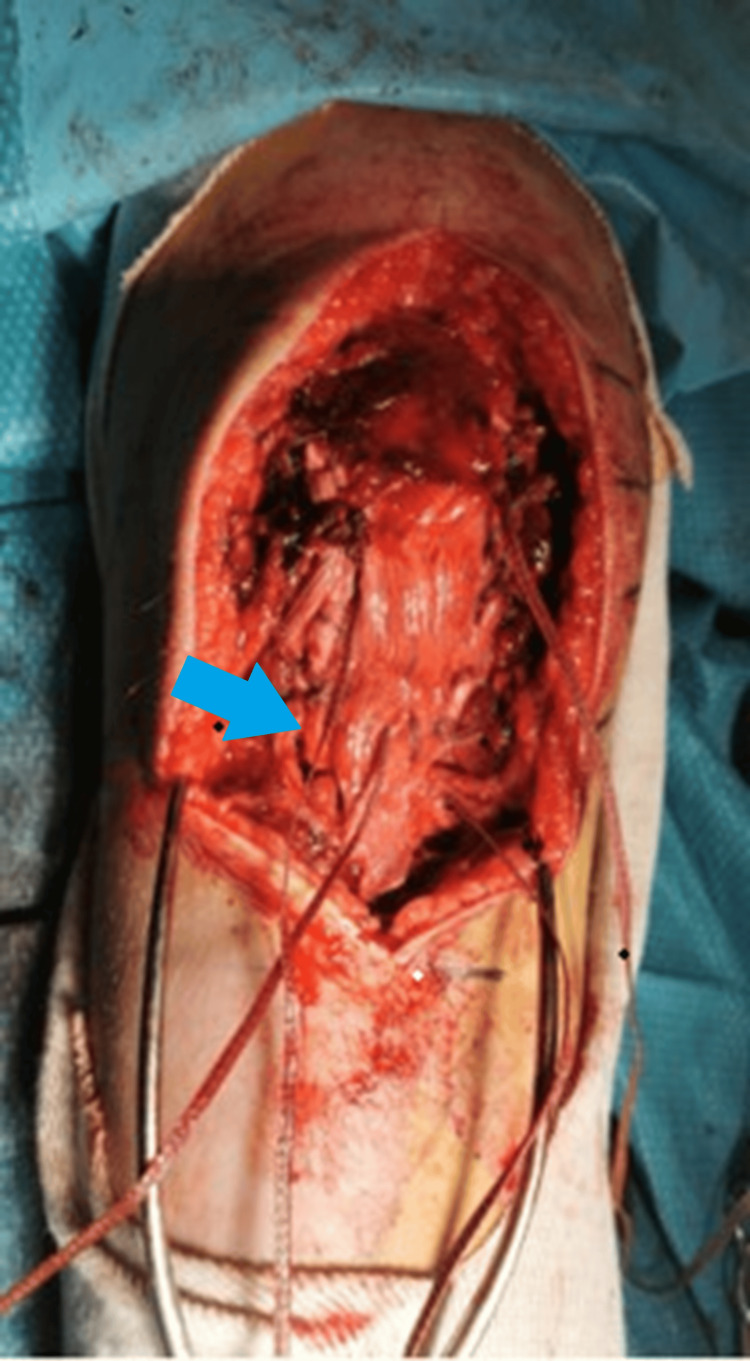
Tendon reattachment using Krakow sutures (blue arrow) and screw augmentation for distal stability.

**Figure 4:**
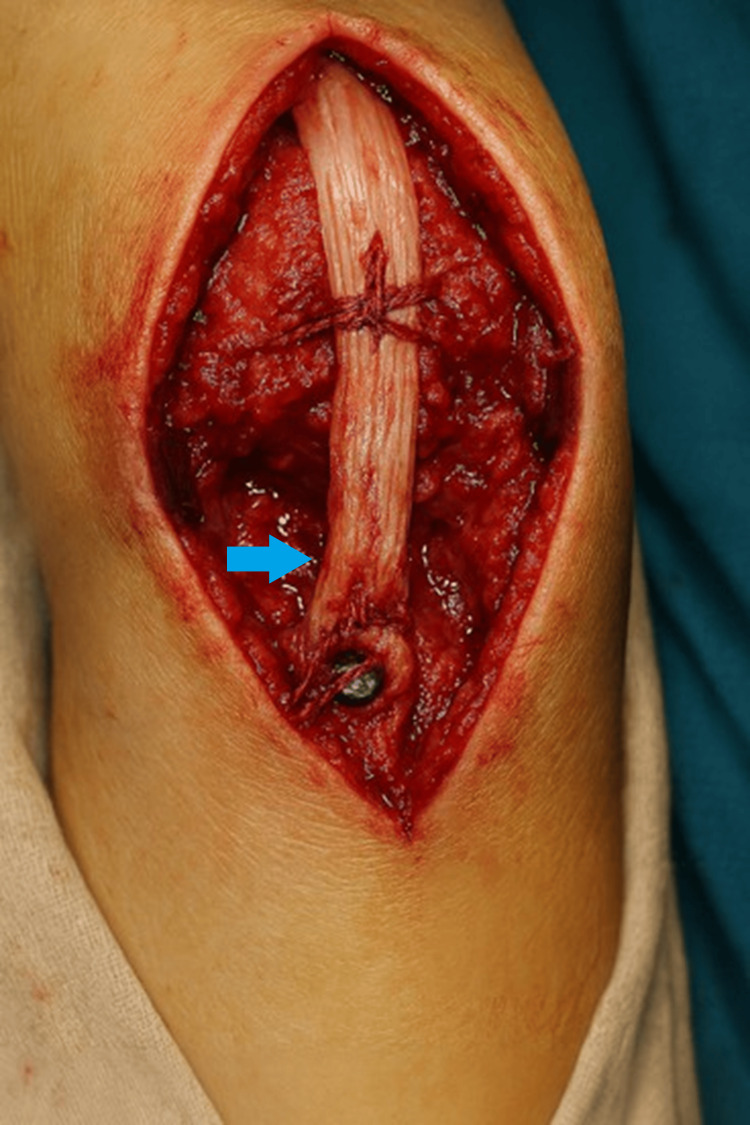
Anatomic repair complete with hardware fixation. The blue arrow shows a repaired patella tendon and a visible screw for tibial tuberosity avulsion repair.

**Figure 5:**
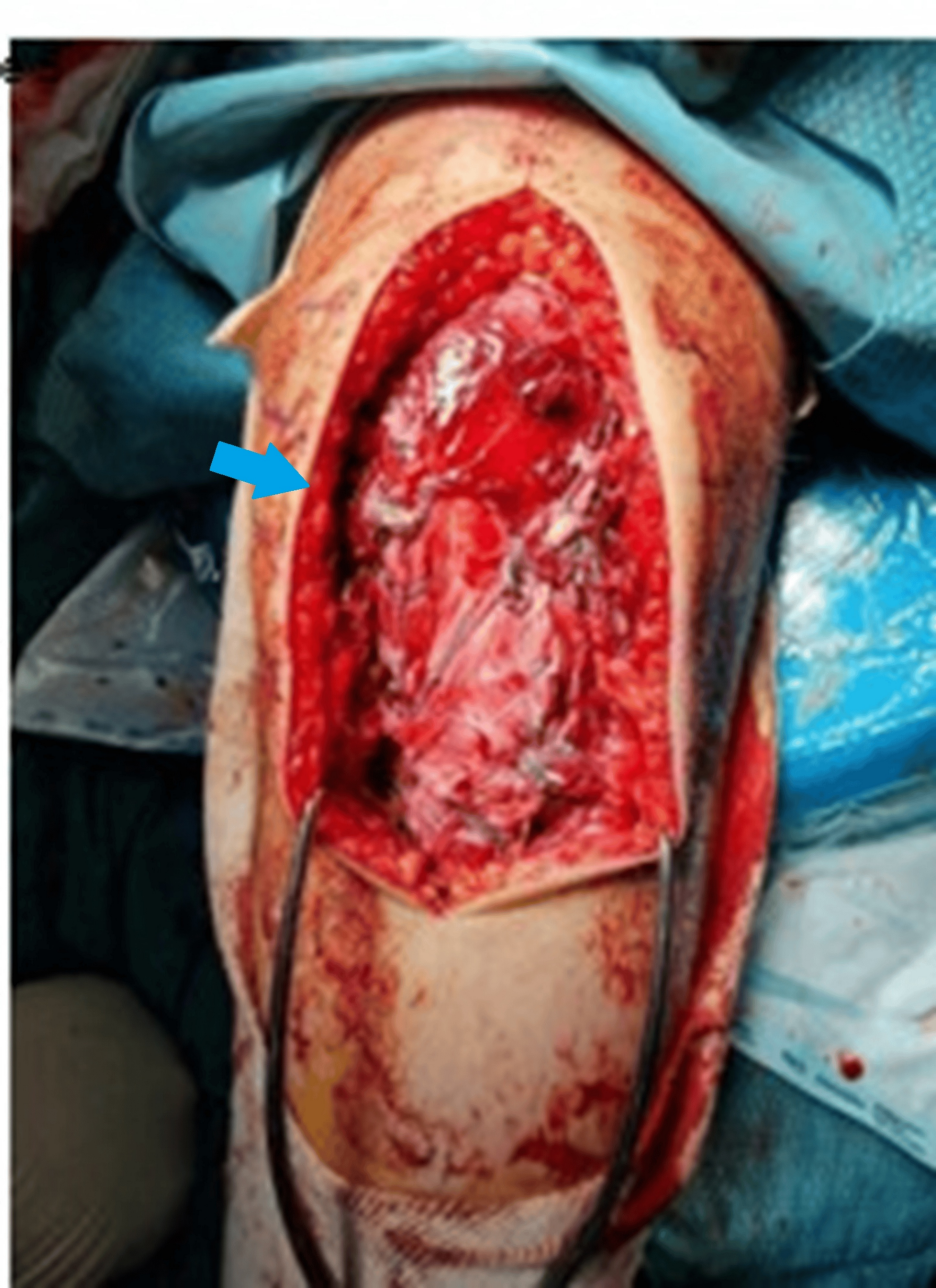
Final construct with repaired retinaculum (blue arrow).

**Figure 6:**
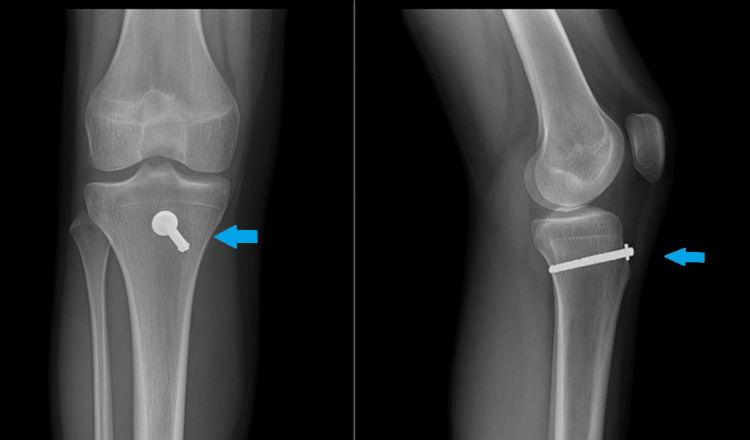
X-ray image at 10-week review. Metalwork was removed at 13 months postoperatively (blue arrow).

Intraoperative findings confirmed the triad: a sleeve fracture at the inferior pole, a distal avulsion at the tibial insertion, and significant retinacular tearing (Figure 2). Surgical stabilization employed modern internal bracing concepts to enable an earlier range of motion (ROM) (Figures 3-5) [11]. For tibial tuberosity avulsion, the repair was further augmented with screw insertion (Figure 6).

Postoperative management followed a structured protocol designed to balance the protection of the surgical construct with the prevention of arthrofibrosis. Follow-up clinical reviews were scheduled at two, six, and 12 weeks, with subsequent assessments at six months and one year to evaluate osseous union and functional performance. Planned removal of the tibial hardware was scheduled at the 13-month mark. Rehabilitation prioritized the early restoration of ROM through a staged approach. During the initial six-week period, the emphasis was placed on passive ROM exercises within a protected range to mitigate joint stiffness while safeguarding the internal bracing. Active knee extension was restricted until radiographic evidence confirmed sufficient healing. At the six-week review, the rehabilitation program progressed to active ROM and full weight-bearing, with specific focus on quadriceps activation and neuromuscular control. Success in this protocol was defined by the achievement of full, pain-free passive and active ROM by the 12-week milestone, followed by progressive closed-chain resistance training and sport-specific functional retraining to ensure symmetry and stability prior to return to contact sports.

## Discussion

The diagnostic challenge presented by this case mirrors the "tip of the iceberg" phenomenon described in the literature, where the cartilaginous nature of the pediatric patella masks the true severity of the displacement [1,2]. While initial radiographs in our case suggested only subtle changes, the intraoperative reality of a massive osteochondral disruption confirms the assertions that MRI is an essential preoperative tool for mapping the full extent of periosteal damage [12,13].

The knee extensor mechanism functions as an integrated kinetic chain comprising the quadriceps muscle, patella, and patellar tendon. It acts as a specialized pulley system, where the patella serves as a fulcrum to significantly enhance the mechanical advantage and lever arm of the quadriceps. This linkage is essential for efficient force transmission from the thigh to the tibia, allowing for lower extremity propulsion. Consequently, disruption at any point along this continuous unit catastrophically impairs the mechanism, resulting in an immediate inability to perform active knee extension.

Our surgical strategy directly addressed the literature's emphasis on anatomical reduction as the primary factor in preserving symmetric patellar growth [2,3]. However, while many traditional protocols advocate for transosseous pull-out sutures, the multilevel instability we encountered involving the patellar sleeve, distal tendon, and retinacula has led us to utilize suture anchors and reinforced augmentation. This approach is supported by recent biomechanical studies suggesting that such constructs provide the superior structural integrity required for high-energy injury patterns in pediatric patients [4,14].

Furthermore, the "triple injury" triad observed in our patient necessitated a more comprehensive repair than the isolated sleeve fractures often described in clinical reports. By performing primary repair of the medial and lateral retinacula, we addressed the need for transverse stability and proper patellar tracking [15]. Finally, our postoperative transition to early, protected motion protocols aligns with modern evidence-based shifts away from traditional casting. Avoiding prolonged immobilization is critical in the pediatric population to prevent permanent joint stiffness and terminal loss of flexion [16].

## Conclusions

This case demonstrates a rare combination of an inferior patellar sleeve fracture, distal tendon avulsion, and bilateral retinacular disruption. The role of imaging modality, like MRI, can prove pivotal in mapping precise surgical repair. Early surgical repair and phased rehabilitation led to functional recovery. Awareness of complex extensor injuries can improve recognition and management in pediatric patients.
